# The Effectiveness of Self-Expandable Metallic Stent Insertion in Treating Right-Sided Colonic Obstruction: A Comparison between SEMS and Decompression Tube Placement and an Investigation of the Safety and Difficulties of SEMS Insertion in Right Colons

**DOI:** 10.1155/2014/372918

**Published:** 2014-12-16

**Authors:** Rintaro Moroi, Katsuya Endo, Ryo Ichikawa, Hiroshi Nagai, Hirohiko Shinkai, Tomoya Kimura, Fumitake Ishiyama, Kei Yaguchi, Shoichi Kayaba, Tooru Shimosegawa

**Affiliations:** ^1^Department of Gastroenterology, Iwate Prefectural Isawa Hospital, 61 Ryugababa, Mizusawa-Ku, Oshu, Iwate 023-0865, Japan; ^2^Division of Gastroenterology, Tohoku University Graduate School of Medicine, 1-1 Seiryo, Aoba-ku, Sendai, Miyagi 980-8574, Japan

## Abstract

*Objectives.* Self-expandable metallic stent (SEMS) is widely used to treat malignant colonic obstruction. However, most reports about SEMS insertion have concentrated on the left colon. This study aimed to (1) investigate the effectiveness of SEMS insertion compared with conventional decompression tube for right-sided colonic obstruction and (2) compare the safety and technical success of SEMS insertion between left- and right-sided colonic obstructions.* Methods.* The data from thirty-seven patients who underwent SEMS or conventional decompression tube placement for malignant colonic obstruction in our hospital were analyzed retrospectively. Technical and clinical success, complications, and technical difficulties were analyzed. We compared the results between SEMS insertion and decompression tube placement in right colons and the outcomes of SEMS insertion between right- and left-sided colonic obstructions.* Results.* For right colons, the clinical success rate of SEMS insertion (100%) was significantly higher than that of decompression tube placement (55.9%). Concerning SEMS insertion, the technical difficulty and safety of SEMS insertion were similar between right- and left-sided colonic obstructions.* Conclusion.* SEMS insertion for right-sided colon is significantly more effective than conventional decompression tube placement, and this procedure was safer and less technically challenging than expected. SEMS insertion should be considered for treating right-sided malignant colonic obstruction.

## 1. Introduction

Colonic obstruction complicates approximately 10–30% of colorectal cancers [1–3]. Treatment strategies for malignant colorectal obstruction include urgent surgery, conventional decompression tube placement (transnasal or transanal), and metallic stent insertion.

Self-expandable metallic stents (SEMSs) are widely used to treat malignant colonic obstruction. SEMSs are used as a bridge to surgery (BTS) and as a palliative therapy.

Obstructive colon cancers in the left colon are recognized as an indication for SEMS insertion. Shimada et al. reported that SEMS insertion into the left colon had several benefits compared to decompression tube placement, such as (1) rapid resolution of obstruction because of the larger internal diameter of SEMSs, (2) the resumption of oral intake, (3) easier management because SEMSs do not require washing, and (4) reduced stress relative to decompression tube placement [4]. Although 40% of obstructive colorectal cancers are located in the right colon (proximal to the splenic flexure) [1], SEMSs are not indicated for right-sided colonic obstruction [5, 6]. Right or extended right hemicolectomy is generally recommended for obstruction above the splenic flexure [5] because it is more difficult to insert SEMSs into the right colon than into the left colon [6]. Furthermore, no differences in the mortality or leakage rates of emergency surgery for obstructive colorectal cancers were noted between patients with right- and left-sided lesions [7]. However, fewer studies have reported on SEMS insertion for right-sided obstructive colon cancer [8, 9]. Mergener and Kozarek reported that fewer than 5% of reported cases of colonic stenting involved the right colon [10]. If we could safely utilize SEMSs for right-sided colonic obstruction, patients would experience fewer surgical complications and improved quality of life (QOL).

This study aimed to (1) investigate the effectiveness of SEMS insertion for right-sided obstructive colon cancer compared to conventional decompression tube placement and (2) confirm the safety and technical success of SEMS insertion in the right colon compared to left-sided SEMS insertion.

## 2. Methods

### 2.1. Patients

From December 2007 to April 2014, 53 patients were admitted to Iwate Prefectural Isawa Hospital because of the malignant colonic obstruction. 16 patients were excluded in this study. Nine patients were treated with no oral intake (partial obstruction) and 7 patients were operated urgently (complete obstruction). The data obtained from another 37 consecutive patients who underwent emergency insertion of a SEMS or decompression tube (transnasal or transanal) for obstructive colorectal cancers were analyzed retrospectively. Their degree of obstruction was complete. They could not take foods at all or had no defecation.

These patients underwent computed tomography (CT) to evaluate the primary lesion and bowel dilatation. Endoscopic examination was also performed to recognize colonic obstruction. Obstructive colorectal cancers were defined as a dilatation proximal to the tumor in the CT scan and denoted by symptoms such as vomiting, abdominal fullness, and abdominal pain. The right colon was defined as the cecum, ascending colon, and transverse colon proximal to the splenic flexure. The left colon was defined as the descending colon distal to the splenic flexure, sigmoid colon, and rectum.

The medical records of each patient were reviewed, and clinical data such as age, sex, and location of the lesion were gathered.

Institutions ethics committee approved this study and the consent of the patients was taken to publish this data.

### 2.2. Devices

Before December 2011, conventional decompression tube placement was used for all cases of obstructive colorectal cancer. If the lesion was located in the right colon, a transnasal approach was selected. If the lesion was located in the left colon, a transanal approach was selected. After January 2012, SEMSs were selected for all patients because SEMS insertion was covered by insurance for malignant bowel obstruction in Japan.

We used 2 types of SEMSs: (1) WallFlex colonic stent (Boston Scientific, Japan) and (2) Niti-S Eternal colonic D-Type stent (TaeWoong Medical, Korea). The through-the-scope technique for stent placement was previously described [11].

We used the colonoscope, PCF-Q240I (Olympus medical system, Japan), for all cases of SEMS placement. The manipulation time of SEMS insertion (from endoscopy to the completion of SEMS placement) was calculated.

### 2.3. Comparison

Technical success was defined as a successful SEMS placement on the first attempt with correct deployment confirmed radiologically. Clinical success was defined as clinical and radiological evidence of relief of obstruction (defecation and loss of niveau or decrease of the colon gas) within 24 h of SEMS insertion.

We compared the results between SEMS and decompression tube placement in the right colon. We also compared the results of SEMS insertion between the right and left colon.

Several factors were compared between right- and left-sided SEMS insertion, including palliation or BTS, stent type and size, technical and clinical success rates, complications, manipulation times, and times to oral intake.

### 2.4. Statistics

Statistical analysis was performed using Fisher's exact test or the Mann-Whitney *U* test. The results were considered significant at *P* < 0.05.

## 3. Results

### 3.1. Overall Result

**Figure 1:**
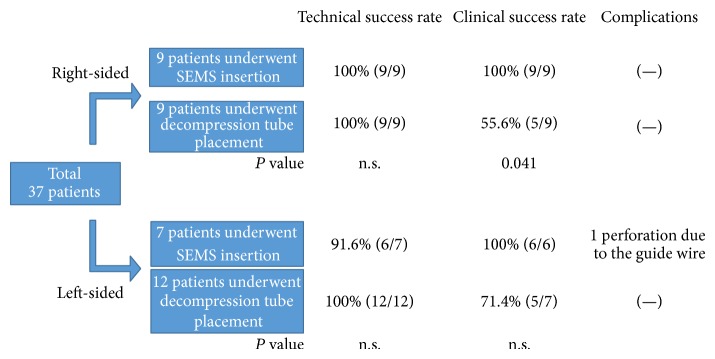
Overall results of this study.

Thirty-seven patients (22 men and 15 women; mean age, 75.4 years) were included in the study (Table 1). The tumor type was colon adenocarcinoma in all but 1 patient (direct invasion from ovarian cancer). Concerning the right colon, 9 patients underwent conventional decompression tube placement, and 9 patients underwent SEMS insertion. Regarding the left colon, 12 patients underwent SEMS insertion, and 7 patients underwent decompression tube placement (Figure 1).

Only one patient (complete obstruction) in the left-sided SEMS group showed a complication, perforation due to the guide wire. Urgent surgery was performed, and no complications were observed after the surgery.

Average follow-up period of BTS cases (time to surgery) was 23.8 days (from 10 days to 146 days) and, similarly, that of palliative cases was 57 days (from 8 days to 133 days).

### 3.2. SEMS Insertion Was Associated with Significantly Better Decompression of Right-Sided Malignant Colonic Obstruction than Conventional Decompression Tube Placement

Both SEMS insertion and decompression tube placement in the right colon were technically successful in all patients. Although the clinical success rate of SEMS insertion was 100%, that of decompression tube placement was 55.6% (*P* = 0.041; Table 2, Figure 1).

Although 4 patients who were treated with decompression tube needed urgent surgery because of the failure of relieving obstruction, no patients who were treated with SEMS needed urgent surgery.

No complications were observed for either treatment strategy. Although none of the patients undergoing decompression tube placement could resume oral intake, oral intake was resumed in all patients who underwent SEMS insertion within an average of 3 days. No complications were observed in the intraoperative and postoperative periods for SEMS insertion as a BTS.

### 3.3. SEMS Insertion Was Linked to a Trend toward Higher Clinical Success Rates than Decompression Tube Placement in the Left Colon

The technical success rate of SEMS insertion in the left colon was 91.6% (11/12, 1 case of perforation due to guide wire), whereas that of decompression tube placement was 100%. No other complications were recognized in these groups. The clinical success rate of SEMS insertion was 100% (11/11), whereas that of decompression tube placement was 71.4%, although the difference was not significant (*P* = 0.13, Table 2).

### 3.4. There Were No Significant Differences in the Technical Success and Complication Rates for SEMS Insertion between the Left and Right Colon

There were no significant differences in the technical success rate between the right and left colon (100% versus 91.6%, *P* = 0.42). Similarly, the clinical success rate (100% versus 100%), complication rate (0% versus 8.4%, *P* = 0.42), manipulation time (40.8 min versus 37.3 min, *P* = 0.70), and time to oral intake (3.0 days versus 3.1 days, *P* = 0.64) were not significantly different for SEMS insertion between the right and left colon (Table 3).

## 4. Discussion

### 4.1. Effectiveness of SEMS Insertion for Right-Sided Colonic Lesions

Regarding the treatment of malignant colonic obstruction, SEMS insertion is indicated for left-sided colonic lesions; therefore, SEMSs are widely used to decompress colonic obstruction for the purpose of BTS or palliation [5, 12–17], as SEMS insertion was associated with lower complication and mortality rates than urgent surgery. As described previously, SEMS insertion into the left colon has several benefits compared to decompression tube placement [4]. Regarding right-sided colonic obstruction, urgent right hemicolectomy or transnasal decompression tube placement is recommended [5, 6] because of the greater technical difficulties and the lower necessity of decompression associated with right-sided colonic obstruction [6]. Therefore, only a few studies assessed SEMS insertion in the treatment of right-sided obstructive colonic cancer [8, 9], although 40% of obstructive colorectal cancers are located in the right colon [1]. Miyata and Yajima reported high technical and clinical success rates (100 and 100%, resp.) and no complications for SEMS insertion into the right colon [8]. Repici et al. also reported high technical and clinical success rates (95 and 85%, resp.) and no complications for SEMS insertion in the treatment of right-sided colonic obstruction [9]. Similarly, our study also identified a high clinical success rate for SEMS insertion in the treatment of right-sided malignant colorectal obstruction. Moreover, we observed statistically higher clinical success rates compared to conventional decompression tube placement. The reasons for the lower clinical success rate of decompression tube placement included the narrow internal diameter of the decompression tube combined with the presence of solid stool in the right colon, resulting in clogging of the tube and a lack of decompression. By contrast, SEMS effectively decompressed right-sided colonic lesions because of its larger internal diameter, which also permitted oral intake. To the best of our knowledge, this is the first report to demonstrate a significantly higher clinical success rate for SEMS insertion compared to conventional decompression tube placement.

### 4.2. Safety and Difficulties of SEMS Insertion

In our study, the overall complication rate was 4.7% (1/21). This result compares favorably with those of previous studies. Only 1 case of perforation due to guide wire insertion (left side) was noted, and other complications such as migration and bleeding were not observed. There were no differences in complication rates between left- and right-sided lesions. Our data also did not identify significant differences in the manipulation time of SEMS placement between the right and left colon (40.8 min versus 37.3 min, *P* = 0.70). Our study indicated that SEMS placement for right-sided colonic lesions may be safer and less technically challenging than anticipated.

### 4.3. Indication of SEMS Insertion for Right-Sided Colonic Obstruction

This study demonstrated that SEMS insertion for right-sided colonic obstructions is superior to decompression tube placement with respect to the clinical success rate. Furthermore, this study also demonstrated that SEMS insertion into the right colon was as safe and stable as that into the left colon. Regarding right-sided colonic obstruction, decompression tubes restrict oral intake, whereas SEMSs permit oral intake, greatly improving patient QOL. In our study, 16 patients underwent SEMS insertion as a BTS. Meanwhile, all patients who underwent SEMS insertion could resume oral intake, and no complications were observed in the postoperative period. At present, decompression tube placement and immediate surgery are generally recommended for treating right-sided colonic obstruction. However, we should positively consider SEMS insertion based on its effectiveness, safety, and ability to improve patient QOL. For example, palliative SEMS insertion may be suitable for patients whose general condition is poor because of its ability to improve QOL and reduce hospital admission.

### 4.4. Disadvantages of SEMS Insertions and Study Limitations

Sabbagh et al. reported worse overall survival after SEMS insertion for patients with left-sided malignant colonic obstruction compared with immediate surgery [18]. The long-term prognosis of SEMS as a BTS is unknown, although the short-term efficacy of this strategy is clear. Moreover, this series of patients was gathered from a single institution, and the number of patients who underwent SEMS insertion was small. Therefore, a study involving a large series of patients with right-sided colonic obstruction who underwent SEMS is necessary to evaluate the long-term prognosis and precise details (e.g., complications and safety) of this strategy.

## 5. Conclusion

Our study revealed that (1) SEMS insertion is more effective than conventional decompression tube for treating right-sided colonic obstruction and (2) the technical difficulties and safety are not different for right-sided SEMS insertion compared to left-sided SEMS insertion.

SEMS insertion appears to be a useful treatment strategy for malignant colonic obstruction even if the lesion is located in the right colon.

## Figures and Tables

**Table 1:** Patient demographics and procedure details of this study.

Number	Age/sex	Obstruction location	Device	Technical result	Clinical result
Left-sided					
1	62/M	Descending colon	Transanal decompression tube	Success	Success
2	65/M	Rectum	Transanal decompression tube	Success	Success
3	72/M	Rectum	Transanal decompression tube	Success	Success
4	75/M	Sigmoid colon	Transanal decompression tube	Success	Success
5	82/F	Sigmoid colon	Transanal decompression tube	Success	Failure
6	74/F	Sigmoid colon	Transanal decompression tube	Success	Success
7	77/M	Sigmoid colon	Transanal decompression tube	Success	Failure
8	80/M	Sigmoid colon	Niti-S 80 × 22 mm, BTS	Success	Success
9	60/M	Rectum	WallFlex 90 × 22 mm, BTS	Success	Success
10	90/F	Rectum	WallFlex 60 × 22 mm, BTS	Success	Success
11	84/F	Descending colon	WallFlex 60 × 22 mm, BTS	Success	Success
12	78/F	Descending colon (ovarian cancer)	WallFlex 60 × 22 mm, BTS	Success	Success
13	79/F	Descending colon	WallFlex 60 × 22 mm, BTS	Success	Success
14	88/F	Sigmoid colon	Perforation due to guide wire	Failure	NA
15	83/F	Sigmoid colon	WallFlex 90 × 22 mm, BTS	Success	Success
16	67/M	Sigmoid colon	WallFlex 60 × 22 mm, BTS	Success	Success
17	59/M	Rectum	WallFlex 60 × 22 mm, palliative	Success	Success
18	75/M	Descending colon	Niti-S 80 × 22 mm, BTS	Success	Success
19	82/M	Sigmoid colon	Niti-S 80 × 22 mm, BTS	Success	Success

Right-sided					
1	56/M	Ascending colon	Transnasal decompression tube	Success	Success
2	75/M	Ascending colon	Transnasal decompression tube	Success	Success
3	74/M	Cecum	Transnasal decompression tube	Success	Success
4	63/F	Ascending colon	Transnasal decompression tube	Success	Success
5	77/M	Cecum	Transnasal decompression tube	Success	Failure
6	78/M	Cecum	Transnasal decompression tube	Success	Failure
7	84/M	Ascending colon	Transnasal decompression tube	Success	Success
8	72/M	Ascending colon	Transnasal decompression tube	Success	Failure
9	69/M	Ascending colon	Transnasal decompression tube	Success	Failure
10	33/F	Splenic flexure	WallFlex 60 × 22 mm, BTS	Success	Success
11	78/M	Transverse colon	WallFlex 60 × 22 mm, BTS	Success	Success
12	58/M	Ascending colon	Niti-S 80 × 22 mm, BTS	Success	Success
13	77/F	Transverse colon	Niti-S 80 × 22 mm, BTS	Success	Success
14	72/F	Ascending colon	Niti-S 80 × 22 mm, BTS	Success	Success
15	54/F	Ascending colon	Niti-S 80 × 22 mm, BTS	Success	Success
16	80/F	Ascending colon	Niti-S 80 × 22 mm, BTS	Success	Success
17	85/M	Transverse colon	Niti-S 80 × 22 mm, palliative	Success	Success
18	81/F	Ascending colon	Niti-S 100 × 22 mm, palliative	Success	Success

BTS: bridge to surgery.

**Table 2:** Comparison of the clinical success rate between SEMS and decompression tube placement.

	Clinical success	Clinical failure	Success rate
Right-sided			
Decompression tube	5	4	55.6%
SEMS	9	0	100%
			*P* = 0.041

Left-sided			
Decompression tube	5	2	71.4%
SEMS	11	0	100%
			*P* = 0.13

SEMS: self-expanding metallic stent.

**Table 3:** Comparison of SEMS insertion between left- and right-sided colonic obstruction.

	Left-sided	Right-sided	*P* value
Technical result	91.6% (11/12)	100% (9/9)	0.42
Clinical result	100% (11/11)	100% (9/9)	n.s.
Complication rate	8.4% (1/12)	0% (0/9)	0.42
Manipulation time (min)	37.3	40.8	0.7
Time to oral intake (days)	3.1	3.0	0.64
Time to surgery (days)	27.9	18.8	0.27

SEMS: self-expanding metallic stent.
